# Development of culture methods capable of culturing a wide range of predominant species of intestinal bacteria

**DOI:** 10.3389/fcimb.2023.1056866

**Published:** 2023-07-13

**Authors:** Rika Hirano, Izumi Nishita, Riho Nakai, Ayaka Bito, Ryunosuke Sasabe, Shin Kurihara

**Affiliations:** ^1^ Host Microbe Interaction Research Laboratory, Faculty of Bioresources and Environmental Sciences, Ishikawa Prefectural University, Nonoichi, Japan; ^2^ Laboratory of Food Immunology, Department of Science and Technology on Food Safety, Faculty of Biology-Oriented Science and Technology, Kindai University, Kinokawa, Japan

**Keywords:** standard medium, predominant intestinal bacteria, culture, GB medium, intestinal bacteria

## Abstract

In recent years, with the development of non-cultivation approaches, it has become evident that intestinal bacteria have a significant impact on human health. However, because one-third of the genes cannot be annotated, it is difficult to elucidate the function of all intestinal bacteria by *in silico* analysis, and it is necessary to study the intestinal bacteria by culturing them. In addition, various media recommended for each individual bacterium have been used for culturing intestinal bacteria; however, the preparation of each medium is complex. To simultaneously culture many bacteria and compare bacterial phenotypes under the same conditions, a medium capable of culturing a wide range of bacteria is needed. In this study, we developed GAM + blood medium (GB medium), which consists of Gifu anaerobic medium containing 5% (v/v) horse blood; it is easy to prepare and it allowed the successful cultivation of 85% of the available predominant species in the human intestinal microbiota.

## 1 Introduction

Animals maintain a complex microbiota in their intestinal lumen, and it is becoming increasingly clear that the intestinal microbiota and health are closely related (Hsiao Elaine et al., 2013; Rosshart et al., 2017; Sharon et al., 2019; Buffington et al., 2021). Therefore, recent research in the field of gut microbes has focused on the function of intestinal microbiota as a community. Next-generation sequencing analysis of DNA and RNA in human feces has been performed since the early 21^st^ century (Shendure and Ji, 2008). These non-cultivation methods have revealed the gene expression profile of the human intestinal microbiota, the catalogue of human intestinal microbial genes, and the predominant species of the intestinal microbiota (Qin et al., 2010; Nishijima et al., 2016). However, there are several undeveloped aspects of the information that can be obtained from next-generation sequencing analysis. Metagenomic gut microbiota analysis uses DNA extracted from feces for next-generation sequencing. However, it has been reported that the analysis of sequencing results vary greatly depending on that the used DNA extraction protocols (Costea et al., 2017). In addition, bias has been reported in amplicon-based library preparation due to sequencing primers (Gohl et al., 2016). Furthermore, even when amino acid sequences are revealed via next-generation sequencing, one-third of the genes cannot be annotated (Chang et al., 2015), and it is difficult to elucidate and regulate the function of the human intestinal microbiota based on their DNA sequences and microbiota composition.

In 2010, the results of genome analysis using next-generation sequencing without cultivation reported 56 genomes that were predominant in the intestines of Europeans (Qin et al., 2010) (**Table 1**). Of the 56 genomes, 45 could be successfully assigned to cultured strains (white column in **Table 1**). For these 45 species, representative strains are available from the culture collections [such as the American Type Culture Collection (ATCC), the German Collection of Microorganisms and Cell Cultures GmbH (DSMZ), and the Japan Collection of Microorganisms (JCM)] (**Table 1**). However, the 11 genomes that could not be assigned to cultured bacteria cannot be investigated using live bacteria (gray rows in **Table 1**). Similarly, the 50 predominant strains in the gut of Japanese individuals have been reported (Nishijima et al., 2016), of which 41 are available (**Table 2**). To stably culture these species and strains, it is recommended to use the medium (**Tables 1**, **2**) designated for each species by the respective distributing institution. However, when several intestinal bacterial taxa are cultured simultaneously, for example, in a 96-well plate, it is necessary to provide different media for each well. This results in greatly increased time, effort, and cost. In addition, different media hamper physiological comparisons among bacteria owing to differences in composition and accurate quantification of bacterial growth because of the presence or absence of precipitation in the media.

**Table 1:** Fifty-six most dominant species in the gut of Europeans (Qin et al., 2010) and their recommended medium.

Occupancy Rank	Frequent microbial genomes (Qin et al., 2010)	Referenced strain	Medium recommended by the distributer	
1	*Bacteroides uniformis*	JCM 5828	GAM	EG
2	*Alistipes putredinis*	JCM 16772	EG	
3	*Parabacteroides merdae*	JCM 9497	EG	
4	*Dorea longicatena*	DSM 13814	DSM medium 104	
5	*Ruminococcus bromii*	ATCC 27255	–	
6	*Bacteroides caccae*	JCM 9498	EG	
7	*Clostridium*			
8	*Bacteroides thetaiotaomicron*	JCM 5827	GAM	
9	*Eubacterium hallii*	ATCC 27751	ATCC Medium1869	ATCC medium 260
10	*Ruminococcus torques*	ATCC 27756	ATCC medium1589	ATCC medium 260
11	*unknown*			
12	*Ruminococcus*			
13	*Faecalibacterium prausnitzii*	JCM 31915	JCM medium 1130	
14	*Ruminococcus lactaris*	ATCC 29176	ATCC medium 1490	ATCC medium 260
15	*Collinsella aerofaciens*	JCM 7790	EG	
16	*Dorea formicigenerans*	ATCC 27755	ATCC medium 158	ATCC medium 260
17	*Bacteroides vulgatus*	JCM 5826	GAM	EG
18	*Roseburia intestinalis*	DSM 14610	DSM medium 1611	
19	*Bacteroides*			
20	*Eubacterium siraeum*	ATCC 29066	ATCC medium1016	
21	*Parabacteroides distasonis*	JCM 5825	GAM	EG
22	*Bacteroides*			
23	*Bacteroides ovatus*	JCM 5824	GAM	EG
24	*Bacteroides*			
25	*Bacteroides*			
26	*Eubacterium rectale*	JCM 17463	JCM medium 465	JCM medium 1130
27	*Bacteroides xylanisolvens*	JCM 15633	EG	JCM medium 461
28	*Coprococcus comes*	ATCC 27758	ATCC medium 1102	ATCC medium 260
29	*Bacteroides*			
30	*Bacteroides*			
31	*Eubacterium ventriosum*	ATCC 27560	ATCC medium 1589	ATCC medium 260
32	*Phocaeicola dorei*	JCM 13471	EG	
33	*Ruminococcus obeum*	DSM 25238	DSM medium104	
34	*Subdoligranulum variabile*	DSM 15176	DSM medium 339a	
35	*Pseudoflavonifractor capillosus*	ATCC 29799	ATCC medium 260	ATCC medium 1490
36	*Streptococcus thermophilus*	JCM 17834	JCM medium 28	JCM medium 13
37	*Clostridium leptum*	ATCC 29065	ATCC medium 2751	ATCC medium 260
38	*Holdemania filiformis*	DSM 12042	DSM medium104	
39	*Bacteroides stercoris*	JCM 9496	EG	
40	*Coprococcus eutactus*	ATCC 27759	ATCC medium1015	ATCC medium 260
41	*Bacteroides*			
42	*Bacteroides eggerthii*	JCM 12986	EG	
43	*Butyrivibrio crossotus*	DSM 2876	DSM medium330	DSM medium78
44	*Bacteroides finegoldii*	JCM 13345	EG	
45	*Parabacteroides johnsonii*	JCM 13406	EG	
46	*Clostridium*			
47	*Clostridium nexile*	ATCC 27757	ATCC medium 1490	ATCC medium 260
48	*Bacteroides pectinophilus*	ATCC 43243	ATCC medium 1547	
49	*Anaerotruncus colihominis*	JCM 15631	EG	JCM medium 676
50	*Ruminococcus gnavus*	ATCC 29149	ATCC medium 158	ATCC medium 260
51	*Bacteroides intestinalis*	JCM 13265	EG	
52	*Bacteroides fragilis*	JCM 11019	EG	
53	*Clostridium asparagiforme*	DSM 15981	DSM medium 104b	
54	*Enterococcus faecalis*	ATCC 700802	ATCC medium 44	
55	*Clostridium scindens*	JCM 6567	EG	
56	*Blautia hansenii*	JCM 14655	JCM medium 676	

If there is more than one recommended medium, a maximum of two are listed. Gray table rows, unidentified genomes at the species level. Orange table cells, medium recommended by the bacterial strain distributor is EG.

**Table 2:** Fifty most dominant species in the gut of Japanese (Nishijima et al., 2016) and their recommended medium.

Occupancy Rank	Frequent metagenomic reads (Nishijima et al., 2016)	Referenced strain	Medium recommended by the distributer	
1	*Blautia wexlerae*	JCM 17041	JCM medium 465	JCM medium 675
2	*Blautia*			
3	*Bifidobacterium longum*	JCM 1217	JCM medium 13	
4	*Bifidobacterium pseudocatenulatum*	JCM 1200	JCM medium 13	
5	*Eubacterium rectale*	ATCC 33656	ATCC medium 1703	ATCC medium 260
6	*Ruminococcus*			
7	*Bifidobacterium adolescentis*	ATCC 15703	ATCC medium 2107	ATCC medium 260
8	*Collinsella*			
9	*Collinsella aerofaciens*	ATCC 25986	ATCC medium 2107	ATCC medium 260
10	*Bacteroides uniformis*	JCM 5828	GAM	JCM medium 13
11	*Anaerostipes hadrus*	DSM 3319	DSM medium 110	DSM medium 78
12	*Dorea longicatena*	DSM 13814	DSM medium 104	
13	*Bacteroides vulgatus*	JCM 5826	GAM	JCM medium 13
14	*Ruminococcus gnavus*	ATCC 29149	ATCC medium 158	ATCC medium 260
15	*Faecalibacterium prausnitzii*	JCM 31915	JCM medium 1130	
16	*Parabacteroides distasonis*	JCM 5825	GAM	JCM medium 13
17	*Faecalibacterium prausnitzii*	JCM 31915	JCM medium 1130	
18	*Dorea formicigenerans*	ATCC 27755	ATCC medium 158	ATCC medium 260
19	*Ruminococcus obeum*	DSM 25238	DSM medium 104	
20	*Ruminococcus torques*	ATCC 27756	ATCC medium 1589	GAM
21	*Faecalibacterium prausnitzii*	JCM 31915	JCM medium 1130	
22	*Bacteroides dorei*	JCM 13471	EG	
23	*Faecalibacterium prausnitzii*	JCM 31915	JCM medium 1130	
24	*Flavonifractor plautii*	ATCC 29863	ATCC medium 1237	
25	*Parabacteroides merdae*	JCM 9497	EG	
26	*Ruminococcus torques*	ATCC 27756	ATCC medium 1589	ATCC medium 260
27	*Roseburia inulinivorans*	JCM 17584	JCM medium 465	
28	*Tyzzerella nexilis*	ATCC 27757	ATCC medium 1490	ATCC medium 260
29	*Ruminococcus*			
30	*Streptococcus salivarius*	JCM 5707	JCM medium 27	JCM medium 70
31	*Eggerthella lenta*	DSM 2243	DSM medium 78	DSM medium 339
32	*Clostridium*			
33	*Bacteroides fragilis*	JCM 11019	EG	
34	*Ruminococcus obeum*	JCM 31340	JCM medium 1130	
35	*Clostridium bolteae*	JCM 12243	EG	
36	*Bilophila wadsworthia*	ATCC 49260	ATCC medium 1490	
37	*Roseburia intestinalis*	DSM 14610	DSM medium 1611	
38	*Clostridium*			
39	*Coprococcus comes*	ATCC 27758	ATCC medium 1102	ATCC medium 260
40	butyrate−producing bacterium			
41	*Clostridium innocuum*	JCM 1292	EG	JCM medium 13
42	*Bacteroides ovatus*	JCM 5824	GAM	EG
43	*Coprococcus catus*	ATCC 27761	ATCC medium 260	
44	*Eubacterium hallii*	ATCC 27751	ATCC medium 1869	ATCC medium 260
45	*Clostridium clostridioforme*	JCM 1291	EG	JCM medium 13
46	*Roseburia hominis*	JCM 17582	JCM medium 465	JCM medium 1130
47	*Clostridiales*			
48	*Firmicutes*			
49	*Bacteroides thetaiotaomicron*	JCM 5827	EG	
50	*Ruminococcus lactaris*	ATCC 29176	ATCC medium 1490	ATCC medium 260

If there is more than one recommended medium, a maximum of two are listed. Gray table rows, unidentified genomes at the bacterial level. Orange table cells, medium recommended by the bacterial strain distributor is EG.

Therefore, we set out to develop a method capable of culturing a large number of species of intestinal bacteria without producing precipitates, and found a method for utilizing Gifu anaerobic medium (GAM) for both pre-culture and main culture (Gotoh et al., 2017). Using this culturing method, 32 of the 44 predominant species of European gut microbiota available at the time were successfully cultured (Gotoh et al., 2017). Using this system, we previously reported five findings. First, we quantified polyamines in the predominant species of the human gut microbiota and reported the existence of many previously unknown metabolic and transport systems for polyamines (Sugiyama et al., 2017). Second, we used our system to screen for the oligosaccharide Gal-β1,4-Rha, which is not utilized by the predominant species of the human gut microbiota and is specifically utilized by bifidobacteria (Hirano et al., 2021). Third, we have also reported a comprehensive analysis of the growth inhibitory activity of medium-chain fatty acids on the predominant species in the gut of Europeans (Matsue et al., 2019). Fourth, we analyzed the effects of micronized “okara” on the growth and metabolic production of the predominant species (Nagano et al., 2020). Fifth, we analyzed phenylethylamine production by the predominant species in the gut of Europeans and found that phenethylamine from gut bacteria stimulated the production of colonic serotonin (Sugiyama et al., 2022). Thus, a system that can grow a wide range of gut microbiota under the same conditions facilitates cross-species comparisons and provides a variety of insights. However, because 32 species represent only 73% of the 44 species, the development of a culture method capable of culturing a wider variety of intestinal bacteria is desired.

Some studies have reported culturing a wide variety of intestinal bacteria using different media. A modified Gifu anaerobic medium (mGAM) is lighter in color and more transparent and is useful in the isolation and cultivation of anaerobic bacteria and in drug susceptibility testing. A total of 45 species commonly occurring within the human population were inoculated into mGAM, and 34 species (76%) were able to grow (Tramontano et al., 2018). However, when bacteria were isolated from human feces using mGAM, 174 genera were detected by 16S rRNA gene analysis and, 48 genera were isolated, suggesting that many genera cannot be cultured using mGAM (Biclot et al., 2022). Although the gut microbiota medium (GMM) (Goodman et al., 2011) is a chemically defined medium, the number of isolated and cultured bacteria is 70% of the genera (Goodman et al., 2011) and 71% of the families (Rettedal et al., 2014) detected in fecal samples, and 33 of the 45 species commonly occurring within the human population (73%) are culturable (Tramontano et al., 2018). Thus, although attempts have been made to culture a wide range of bacteria, it is difficult to completely represent the gut microbiota.

In this study, we developed a culture medium and method that allows the cultivation of more intestinal bacteria, enables comprehensive and simple cultivation of the predominant species of human gut microbiota, and simplifies the subsequent analysis.

## 2 Materials and methods

### 2.1 Microbe strains

Bacteria were obtained from the American Type Culture Collection (ATCC), the German Collection of Microorganisms and Cell Cultures GmbH (DSMZ), and the Japan Collection of Microorganisms (JCM) (**Table 3**). Bacteria were cultured at 37°C in an anaerobic chamber (10% CO_2_, 10% H_2_, and 80% N_2_; InvivO_2_ 400; Ruskinn Technology, Bridgend, UK).

**Table 3:** Bacterial strains used in this study.

Occupancy Rank				
European (Qin et al., 2010)	Japanese (Nishijima et al., 2016)	Bacterial species	Strain	Tested in
1	10	*Bacteroides uniformis*	JCM 5828^T^	**Figures 1** and **2**
2		*Alistipes putredinis*	JCM 16772^T^	**Figures 1** and **2**
3	25	*Parabacteroides merdae*	JCM 9497^T^	**Figures 1** and **2**
4	12	*Dorea longicatena*	DSM 13814^T^	**Figures 1** and **2**
5		*Ruminococcus bromii*	ATCC 27255^T^	**Figures 1** and **2**
6		*Bacteroides caccae*	JCM 9498^T^	**Figures 1** and **2**
8	49	*Bacteroides thetaiotaomicron*	JCM 5827^T^	**Figures 1** and **2**
9	44	*Eubacterium hallii*	ATCC 27751^T^	**Figures 1** and **2**
10	20, 26	*Ruminococcus torques*	ATCC 27756^T^	**Figures 1** and **2**
13	15, 17, 21, 23	*Faecalibacterium prausnitzii*	JCM 31915	**Figures 1** and **2**
14	50	*Ruminococcus lactaris*	ATCC 29176^T^	**Figures 1** and **2**
15	9	*Collinsella aerofaciens*	JCM 7790	**Figures 1** and **2**
16	18	*Dorea formicigenerans*	ATCC 27755^T^	**Figures 1** and **2**
17	13	*Bacteroides vulgatus*	JCM 5826^T^	**Figures 1** and **2**
18	37	*Roseburia intestinalis*	DSM 14610^T^	**Figures 1** and **2**
20		*Eubacterium siraeum*	ATCC 29066^T^	**Figures 1** and **2**
21	16	*Parabacteroides distasonis*	JCM 5825^T^	**Figures 1** and **2**
23	42	*Bacteroides ovatus*	JCM 5824^T^	**Figures 1** and **2**
26	5	*Eubacterium rectale*	JCM 17463	**Figures 1** and **2**
27		*Bacteroides xylanisolvens*	JCM 15633^T^	**Figures 1** and **2**
28	39	*Coprococcus comes*	ATCC 27758^T^	**Figures 1** and **2**
31		*Eubacterium ventriosum*	ATCC 27560^T^	**Figures 1** and **2**
32	22	*Phocaeicola dorei*	JCM 13471^T^	**Figures 1** and **2**
33	19, 34	*Ruminococcus obeum*	DSM 25238^T^	**Figures 1** and **2**
34		*Subdoligranulum variabile*	DSM 15176^T^	**Figures 1** and **2**
35		*Pseudoflavonifractor capillosus*	ATCC 29799^T^	**Figures 1** and **2**
36		*Streptococcus thermophilus*	JCM 17834^T^	**Figures 1** and **2**
37		*Clostridium leptum*	ATCC 29065^T^	**Figures 1** and **2**
38		*Holdemania filiformis*	DSM 12042^T^	**Figures 1** and **2**
39		*Bacteroides stercoris*	JCM 9496^T^	**Figures 1** and **2**
40		*Coprococcus eutactus*	ATCC 27759^T^	**Figures 1** and **2**
42		*Bacteroides eggerthii*	JCM 12986^T^	**Figures 1** and **2**
43		*Butyrivibrio crossotus*	DSM 2876^T^	**Figures 1** and **2**
44		*Bacteroides finegoldii*	JCM 13345^T^	**Figures 1** and **2**
45		*Parabacteroides johnsonii*	JCM 13406^T^	**Figures 1** and **2**
47	28	*Clostridium nexile*	ATCC 27757^T^	**Figures 1** and **2**
48		*Bacteroides pectinophilus*	ATCC 43243^T^	**Figures 1** and **2**
49		*Anaerotruncus colihominis*	JCM 15631^T^	**Figures 1** and **2**
50	14	*Ruminococcus gnavus*	ATCC 29149^T^	**Figures 1** and **2**
51		*Bacteroides intestinalis*	JCM 13265	**Figures 1** and **2**
52	33	*Bacteroides fragilis*	JCM 11019^T^	**Figures 1** and **2**
53		*Clostridium asparagiforme*	DSM 15981^T^	**Figures 1** and **2**
54		*Enterococcus faecalis*	ATCC 700802	**Figures 1** and **2**
55		*Clostridium scindens*	JCM 6567^T^	**Figures 1** and **2**
56		*Blautia hansenii*	JCM 14655^T^	**Figures 1** and **2**
	1	*Blautia wexlerae*	JCM 17041^T^	**Figure 3**
	3	*Bifidobacterium longum*	JCM 1217^T^	**Figure 3**
	4	*Bifidobacterium pseudocatenulatum*	JCM 1200^T^	**Figure 3**
	7	*Bifidobacterium adolescentis*	JCM 1275^T^	**Figure 3**
	11	*Anaerostipes hadrus*	DSM 3319^T^	**Figure 3**
	24	*Flavonifractor plautii*	ATCC 29863^T^	**Figure 3**
	27	*Roseburia inulinivorans*	DSM 16841^T^	**Figure 3**
	30	*Streptococcus salivarius*	JCM 5707^T^	**Figure 3**
	31	*Eggerthella lenta*	DSM 2243^T^	**Figure 3**
	35	*Clostridium bolteae*	JCM 12243^T^	**Figure 3**
	36	*Bilophila wadsworthia*	ATCC 49260^T^	**Figure 3**
	41	*Clostridium innocuum*	JCM 1292^T^	**Figure 3**
	43	*Coprococcus catus*	ATCC 27761^T^	**Figure 3**
	45	*Clostridium clostridioforme*	JCM 1291^T^	**Figure 3**
	46	*Roseburia hominis*	JCM 17582^T^	**Figure 3**

### 2.2 Preparation of GAM + Eggerth–Gagnon medium (GE)

GAM (Nissui Pharmaceutical, Tokyo, Japan) was autoclaved (115°C for 15 min), immediately placed in a closed container with Aneropack Kenki (Mitsubishi Gas Chemical Company, Tokyo, Japan), and allowed to stand overnight to remove oxygen. Eggerth–Gagnon (EG) medium (composition: proteose peptone No. 3, yeast extract, Na_2_HPO_4_, glucose, soluble starch, l-cystine, l-cysteine ·HCl·H_2_O, and horse blood) was prepared according to the JCM’s instructions^1^. Materials other than blood were autoclaved, placed in a closed container together with Aneropack Kenki, and allowed to stand overnight to remove dissolved oxygen. Horse blood (horse whole blood defibrinated sterile; Nippon Bio-Supp. Center, Tokyo, Japan) stored anaerobically with Aneropack Kenki was added to the GAM at 5% (v/v) in an anaerobic chamber. GAM and EG medium were mixed in a 1:1 (v/v) ratio.

### 2.3 Preparation of GAM supplemented with Blood medium (GB)

GAM was autoclaved (115°C, 15 min), immediately placed in a closed container together with Aneropack Kenki, and allowed to stand overnight to remove dissolved oxygen. Horse blood that was stored anaerobically with Aneropack Kenki was then added to GAM at 5% (v/v) in an anaerobic chamber. To prepare GB_sheep_, sheep blood (Japan Bio Serum, Tokyo, Japan) was added instead of horse blood using the same procedure, and for GB_human_, human blood (Tennessee Blood Services, Tennessee, US) was added instead of horse blood, using the same procedure.

### 2.4 Culturing system

**Figure 1:**
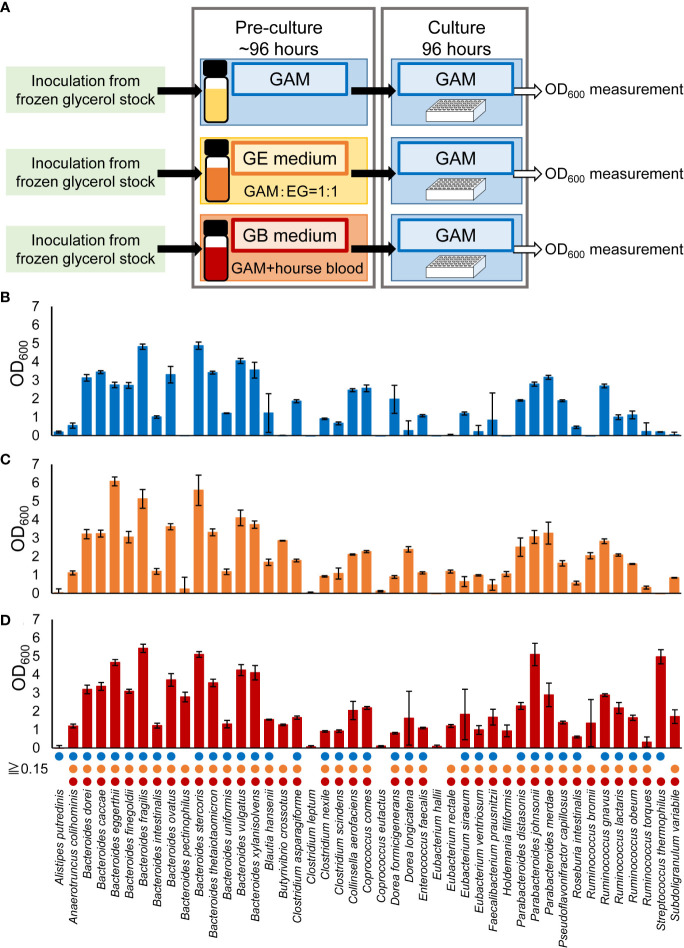
Comparison of pre-culture medium conditions required for culturing a wide range of predominant species of intestinal bacteria. **(A)** Experimental overview. **(B–D)** After pre-culturing in GAM (blue bars in **B**), GE medium (orange bars in **C**), or GB medium (red bars in **D**), the bacterial species were cultured in GAM for 96 hours. Bacterial growth was measured by determining the OD_600_. Circles indicate bacteria with an OD_600_ ≥ 0.15. Data are presented as the mean ± standard deviation (n = 3). **(B)** The growth was confirmed using 16S rDNA sequencing in cases where bacteria that did not grow in previous reports (Gotoh et al., 2017) using the GAM in pre-culture were grown in this study (**Supplementary Table S1**).

**Figure 2:**
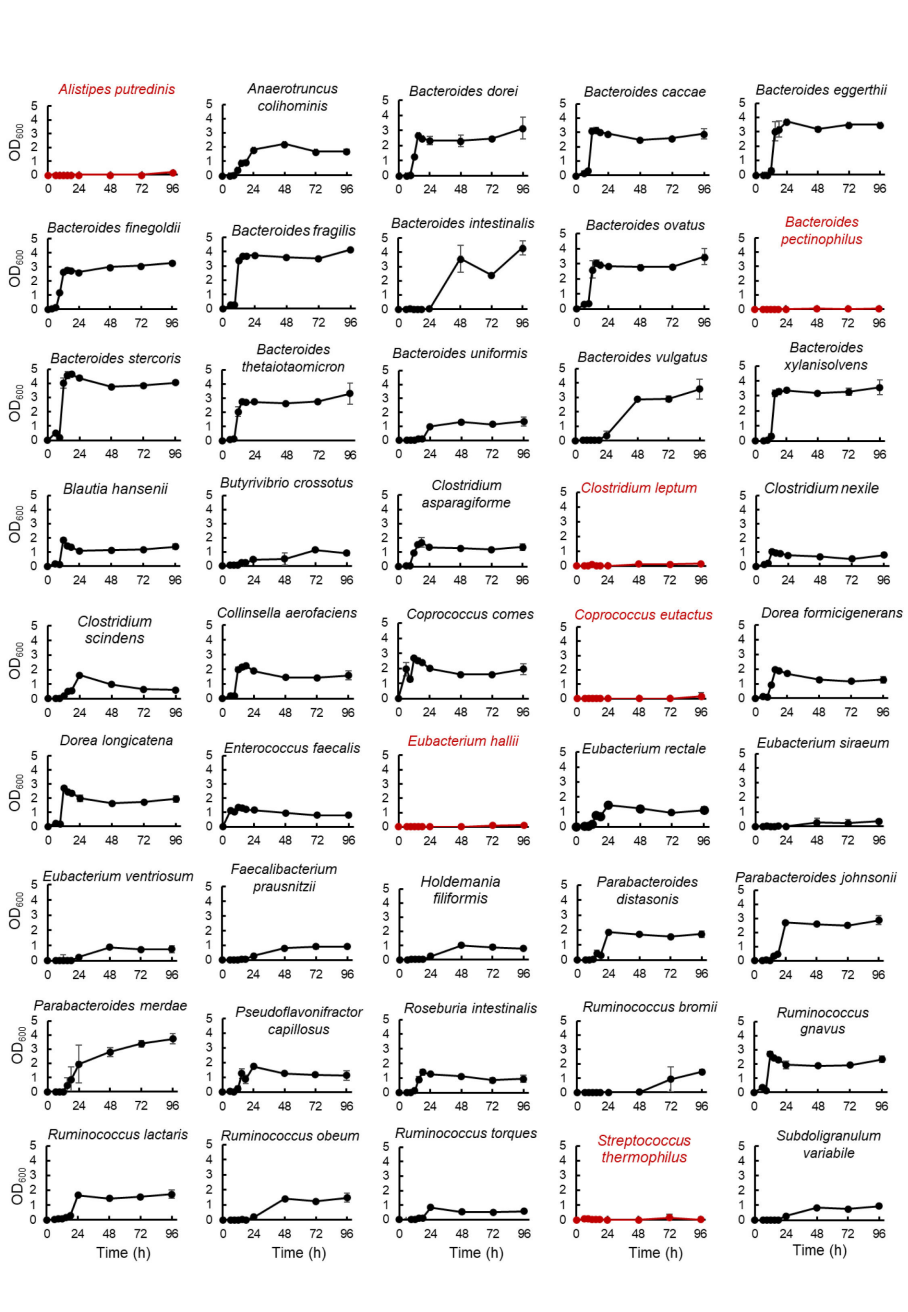
Growth curve of predominant species of intestinal bacteria grown using the developed culture system. After pre-culturing in GB medium, bacterial species were cultured in GAM for 96 hours. Growth was tracked by measuring OD_600_ over time. Bacteria with two or more points with an OD_600_ greater than 0.15 are shown on the graph in black. The growth of these bacteria was confirmed using 16S rDNA sequencing (**Supplementary Table S2**). Bacteria with one or zero points with an OD greater than 0.15 are indicated by red graphs. Data are presented as the mean ± standard deviation (n = 3).

**Figure 3:**
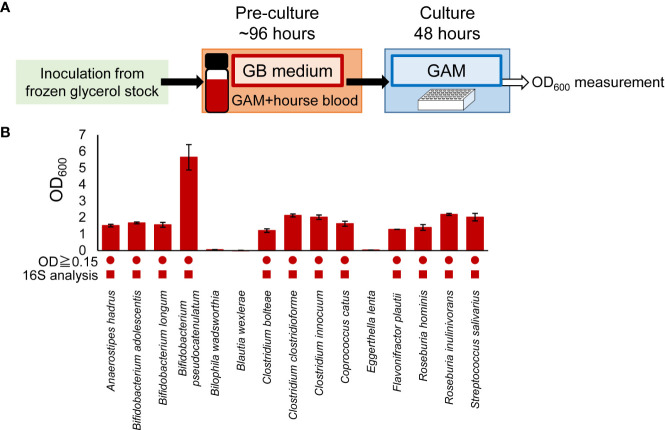
Adaptability of GB medium for predominant species in other gut microbiome projects. **(A)** Experimental overview. **(B)** After pre-culturing in GB medium, bacterial species were cultured in GAM for 48 hours. Bacterial growth was measured by determining the OD_600_. Circles indicate bacteria OD_600_ ≥ 0.15. Square symbols indicate growth confirmed by 16S rDNA sequencing analysis (**Supplementary Table S2**). Data represent means ± standard deviation (n = 3).

The experimental procedure is shown in **Figure 1**. Bacteria were cultured in an anaerobic chamber. First, bacterial strains were inoculated from frozen glycerol stock in 500 μL or 3 mL of media in 96-deep well plates or vials, respectively, and incubated at 37°C for 24-96 hours. GAM (**Figure 1B**), GE (**Figure 1C**) or GB (**Figures 1D**, **2**, **3**, **Supplementary Figures S1**, **S2**) were used as the medium for pre-culture. For pre-culturing in vials, 500 µL of the pre-culture solution was transferred to a 96-well plate before using a copy stand. Approximately 2 µL of the respective culture collection was inoculated in 500 μL of GAM in 96-deep well plates using a copy plate stand (Tokken, Chiba, Japan). After 48 hours (**Figure 3**; **Supplementary Figures S1**, **S2**) or 96 hours (**Figure 1**) of anaerobic incubation, growth was measured as the optical density at 600 nm (OD_600_) using Thermo Scientific™ Multiskan™ GO (Thermo Fisher Scientific, Waltham, MA). For **Figure 2**, measurements were taken over time up to 96 hours. The possibility of culture contamination was eliminated by 16S rDNA sequencing using previously described procedures (Gotoh et al., 2017) (**Supplementary Tables S1, S2**).

## 3 Results

### 3.1 Development of culture media capable of culturing a wide range of bacteria

We previously reported that 32 of the 56 predominant species in the human gut microbiota can be cultured in GAM (Gotoh et al., 2017). As with GAM, EG medium is recommended for numerous gut microbes (**Table 1**). Therefore, GE medium, a 1:1 (v/v) mixture of GAM and EG, was prepared. GAM + blood medium (GB medium) was also prepared by adding 5% (v/v) of horse blood to GAM, with reference to the fact that horse blood was supplemented to the EG medium at a final concentration of 5% (v/v). Of the 56 predominant species of European intestinal commensal microbiota identified using non-culture methods, 45 species available from the culture collection were pre-cultured in GAM, GE, and GB (**Figure 1A**). It was difficult to measure OD_600_ in GE and GB because of the turbidity derived from the added horse blood; therefore, the pre-culture was inoculated into GAM and cultivated anaerobically for 96 h at 37°C to test the growth of bacterial species by measuring the OD_600_ value (**Figure 1A**). The presence or absence of growth was determined using a threshold of OD_600_ = 0.15, as previously described (Tramontano et al., 2018). The number of bacterial species whose growth in GAM exceeded 0.15 was 36 (80% of the tested strains) when the pre-culture was performed on GAM (**Figure 1B**), 40 (89% of the tested strains) when the pre-culture was performed on GE medium (**Figure 1C**), and 41 (91% of the tested strains) when the pre-culture was performed on GB medium (**Figure 1D**). Compared to pre-culture using the conventional method of GAM (Gotoh et al., 2017), the number of species that could be grown was increased using our newly prepared GE or GB media for pre-culture. GB was chosen for subsequent experiments because it was able to culture the greatest number of species.

### 3.2 Stable growth of a wide range of bacteria using GB medium

To verify the stability of the culture of 45 bacterial species, which were confirmed to be growing when pre-cultured in GB and primarily cultured in GAM (**Figure 1D**), the same culturing method was used to culture these 45 bacterial species and measure their growth over time for 96 hours (**Figure 2**). A total of 39 species showed continuous growth (**Figure 2**), and 16S rDNA analysis of the bacterial cultures confirmed the species of the growing bacteria (**Supplementary Table S2**). Four species that did not grow in the conditions described in **Figure 1D** (*Alistipes putredinis*, *Eubacterium hallii*, *Clostridium leptum*, and *Coprococcus eutactus*) also did not grow in the conditions described in **Figure 2**. *Streptococcus thermophilus* and *Bacteroides pectinophilus* grew in the conditions described in **Figure 1D**; however, continuous growth was unstable (**Figure 2**). Thus, 39 of the 45 (87%) available species of the predominant species of European intestinal microbiota can be stably cultured using GB for pre-culture and GAM for the main culture.

### 3.3 Application of culture methods using GB medium to gut microbiota most dominant species derived from different human populations

It is becoming clear that the predominant species of bacteria vary in different human populations. Therefore, to investigate whether the culturing methods developed in this study could be applied to the predominant species in other gut microbiome projects, we cultured the predominant species of intestinal microbiota in the Japanese population (Nishijima et al., 2016) using GB for pre-culture and GAM for main culture (**Figure 3**). The predominant species of intestinal microbiota in the Japanese population were as diverse as the predominant species in Europeans (**Table 2**). As with Europeans, there was also a wide variety of recommended media (**Table 2**). Of the 50 predominant species of intestinal microbiota in Japanese individuals, 41 species publicly available from distributors such as the JCM, the ATCC, and the DSMZ were selected for examination (**Table 3**). Twenty-six strains were excluded from the study because they were identical to the predominant species in Europeans (**Table 3**). Consequently, 15 bacterial species (**Table 3**) were newly cultured in GB for pre-culture and GAM for the main culture (**Figure 3**). Because most predominant bacterial species of the European gut microbiota reached the stationary phase at 48 h of culture (**Figure 2**), the culture was not cultivated further (**Figure 3A**). Of the 15 strains, 12 grew sufficiently with an OD_600_ greater than 0.15, and contamination of the culture was excluded by 16S rDNA sequencing (**Figure 3B** and **Supplementary Table S2**). Together with the results of **Figure 2**, 32 of the 36 species (89%) intestinal microbiota in Japanese were cultured. These results indicate that GB is a potential medium for growing a wide range of bacterial species, the existence of which has been suggested in numerous human gut microbiome projects without culturing.

### 3.4 Effect of replacement of horse blood with other mammal’s blood on bacterial growth

Next, growth was tested when the horse blood added to the GB medium was replaced by blood from other mammals. Fifty-one species grown in GB medium containing horse blood were cultured in GB_sheep_ or GB_human_ medium prepared using sheep or human blood, respectively, instead of horse blood. The results show that 48 (94% of the tested 51 strains successfully cultured in GB) and 45 (88% of the tested 51 strains successfully cultured in GB) strains grew in GB_sheep_ (**Supplementary Figure S1**) and GB_human_ (**Supplementary Figure S2**), respectively.

## 4 Discussion

In this study, we succeeded in developing a new method for culturing a wide range of intestinal bacteria under the same conditions using an easily prepared GB medium, which can be prepared from only two materials thereby reducing the time and effort required for culturing. Using GB medium, 51 of 60 strains (85%) of European- and Japanese-predominant species were successfully cultured. Some of the predominant species, such as *Subdoligranulum variabile* and *Roseburia hominis*, which were previously unculturable in GMM, mGAM, or GAM, were cultured in GB (**Table 4**).

**Table 4:** Growth of the most dominant species in mGAM, GMM, GAM and GB.

Occupancy Rank			mGAM	GMM	GAM	GB
European (Qin et al., 2010)	Japanese (Nishijima et al., 2016)	Species	(Tramontano et al., 2018)	(Tramontano et al., 2018)	(Gotoh et al., 2017)	This study
1	10	*Bacteroides uniformis*	+	+	+	+
2		*Alistipes putredinis*	−	−	−	−
3	25	*Parabacteroides merdae*	+	+	+	+
4	12	*Dorea longicatena*	n/a	n/a	+	+
5		*Ruminococcus bromii*	+	−	−	+
6		*Bacteroides caccae*	+	+	+	+
8	49	*Bacteroides thetaiotaomicron*	+	+	+	+
9	44	*Eubacterium hallii*	n/a	n/a	−	−
10	20, 26	*Ruminococcus torques*	−	+	+	+
13	15, 17, 21, 23	*Faecalibacterium prausnitzii*	n/a	n/a	n/a	+
14	50	*Ruminococcus lactaris*	n/a	n/a	+	+
15	9	*Collinsella aerofaciens*	+	+	+	+
16	18	*Dorea formicigenerans*	+	+	+	+
17	13	*Bacteroides vulgatus*	+	+	+	+
18	37	*Roseburia intestinalis*	+	+	+	+
20		*Eubacterium siraeum*	+	+	+	+
21	16	*Parabacteroides distasonis*	+	+	+	+
23	42	*Bacteroides ovatus*	+	+	+	+
26	5	*Eubacterium rectale*	+	+	−	+
27		*Bacteroides xylanisolvens*	+	+	+	+
28	39	*Coprococcus comes*	+	+	+	+
31		*Eubacterium ventriosum*	n/a	n/a	+	+
32	22	*Phocaeicola dorei*	+	+	+	+
33	19, 34	*Ruminococcus obeum*	+	+	+	+
34		*Subdoligranulum variabile*	n/a	n/a	−	+
35		*Pseudoflavonifractor capillosus*	n/a	n/a	+	+
36		*Streptococcus thermophilus*	n/a	n/a	−	−
37		*Clostridium leptum*	−	−	−	−
38		*Holdemania filiformis*	n/a	n/a	−	+
39		*Bacteroides stercoris*	+	+	+	+
40		*Coprococcus eutactus*	n/a	n/a	−	−
42		*Bacteroides eggerthii*	+	+	−	+
43		*Butyrivibrio crossotus*	+	−	−	+
44		*Bacteroides finegoldii*	n/a	n/a	+	+
45		*Parabacteroides johnsonii*	n/a	n/a	+	+
47	28	*Clostridium nexile*	n/a	n/a	+	+
48		*Bacteroides pectinophilus*	n/a	n/a	−	−
49		*Anaerotruncus colihominis*	n/a	n/a	+	+
50	14	*Ruminococcus gnavus*	+	+	+	+
51		*Bacteroides intestinalis*	n/a	n/a	+	+
52	33	*Bacteroides fragilis*	+	+	+	+
53		*Clostridium asparagiforme*	n/a	n/a	+	+
54		*Enterococcus faecalis*	n/a	n/a	+	+
55		*Clostridium scindens*	n/a	n/a	+	+
56		*Blautia hansenii*	+	+	+	+
	1	*Blautia wexlerae*	n/a	n/a	n/a	−
	3	*Bifidobacterium longum*	+	+	n/a	+
	4	*Bifidobacterium pseudocatenulatum*	n/a	n/a	n/a	+
	7	*Bifidobacterium adolescentis*	+	+	n/a	+
	11	*Anaerostipes hadrus*	n/a	n/a	n/a	+
	24	*Flavonifractor plautii*	n/a	n/a	n/a	+
	27	*Roseburia inulinivorans*	n/a	n/a	n/a	+
	30	*Streptococcus salivarius*	+	+	n/a	+
	31	*Eggerthella lenta*	n/a	n/a	n/a	−
	35	*Clostridium bolteae*	+	+	n/a	+
	36	*Bilophila wadsworthia*	−	−	n/a	−
	41	*Clostridium innocuum*	n/a	n/a	n/a	+
	43	*Coprococcus catus*	n/a	n/a	n/a	+
	45	*Clostridium clostridioforme*	n/a	n/a	n/a	+
	46	*Roseburia hominis*	−	−	n/a	+

n/a, there were no description about growth in reference.

We have cultured *Flavonifractor plautii* many times using this system, but the cultivation is not always successful. In this report, we have provided data from a successful culture. There is a need for further improved culturing methods for better reproducibility.

In this study, we selected and cultured representative strains of each species. Bacterial strains, even those of the same species, vary in their characteristics, and these differences may affect human health (Yan et al., 2020). Since it is unclear whether other strains of the same species can be cultured using the method described in this study, it needs to be attempted in the future.

In addition, we have yet to attempt to isolate bacteria from feces using the GB medium. Additional experiments are needed to use the methods described in this study for the isolation of unknown bacteria from human feces. In the future, we plan to determine how many of the fecal bacteria (as detected from fecal DNA information by non-cultivation approaches) can be isolated using GB media.

A liquid growth medium was used in this study to simultaneously culture many bacterial species at the same time. It is difficult to simultaneously culture dozens of different bacteria on solid media because of the large space required for culturing. However, culturing on solid media is necessary to isolate bacteria. Moreover, cell growth can be directly confirmed by colony formation when cultured on solid media.

In a previous report, in which GAM was used in the pre-culture and GAM in the main culture, 32 species were grown (Gotoh et al., 2017). In this study, 36 species were successfully grown (**Figure 1B**). This may be attributed to the extended incubation time of the main culture up to 96 h (this study) compared to the previous 48 h (previous report).

Remarkably, the number of culturable species increased when GB was used in the pre-culture (**Figure 1D**) compared to when GAM was used (**Figure 1B**), even though the main culture had the same GAM. In this culture system, approximately 2 µL was brought into the main culture from the pre-culture medium, which was only 0.4% of the volume. In bacterial culture, it is suggested that if the pre-culture is carefully devised, the subsequent successional culture can grow well, even if the medium and bacteria are somewhat nutritionally incompatible. In the food industry, starter culture, which is equivalent to pre-culture, is used in the production of fermented foods. Starter culture may be defined as “a preparation or material containing large numbers of variable microorganisms, which may be added to accelerate a fermentation process” (Holzapfel, 2002). Starter cultures are used to manufacture foods such as cheese (Somerville et al., 2022), yogurt (Chen et al., 2017), sake (Yamashita, 2021), and wine (Capozzi et al., 2015), for example. Although initiation of spontaneous fermentation requires a relatively long time, using a starter culture can shorten this time (Holzapfel, 2002). The pre-culture may be used to improve subsequent growth. Indeed, it has been reported that two-stage cultures, including a pre-culture to promote growth, were used to isolate bacteria from feces and helped successfully culture a multitude of new species (Lagier et al., 2016). Thus, pre-culturing is an important step for bacterial analysis *via* culturing.

Notably, in this study, we successfully cultured *Faecalibacterium prausnitzii* JCM 31915. *F. prausnitzii* is reduced in the gut microbiota of donors with type 2 diabetes (Qin et al., 2012), Crohn’s disease (Fujimoto et al., 2013) and cirrhosis (Qin et al., 2014) compared to healthy donors. *F. prausnitzii* is very difficult to culture, and the preparation of the recommended medium, JCM 1130 medium (YCFA medium), requires a mixture of more than 20 ingredients. It has also been reported that *F. prausnitzii* can be cultured in mGAM-CRI medium, which is prepared by supplementing mGAM with bovine rumen, cellobiose, and inulin (Bellais et al., 2022). In this study, we found that *F. prausnitzii* JCM 31915 could be cultured on GB medium, which is more easily prepared and has fewer ingredients than other media. In addition, several phylogenetic groups exist in *F. prausnitzii* and have recently been reclassified into the following four species (Sakamoto et al., 2022): *F. prausnitzii* (type strain ATCC 27768^T^), *Faecalibacterium duncaniae* (type strain JCM 31915^T^ tested in this study), *Faecalibacterium hattorii* (type strain JCM 39210^T^), and *Faecalibacterium gallinarum* (type strain JCM 17207^T^). Using our method, it may be possible to culture three other species (*F. prausnitzii*, *F. hattorii*, and *F. gallinarum*).

It has been estimated that there are more than 1,000 uncultured bacterial species in the human gut based on metagenomic analysis (Almeida et al., 2019). Our culturing method using GB medium, which is easy to prepare, may be applicable to the culture of bacteria whose functions and ecology are unknown and should be tested in the future. Culturomics, a culturing approach using bacterial culture, MALDI-TOF mass spectrometry, and 16S rRNA sequencing, have been developed for the cultivation and identification of unknown bacteria (Lagier et al., 2018). In culturomics, there are reports of successful analysis of new bacterial species by improving the culture medium (Lagier et al., 2012; Lagier et al., 2016). Furthermore, the use of GB media, combined with techniques such as culturomics, would also help in the analysis of unknown bacteria.

Administration of antibiotics and prebiotics can significantly modify the gut microbiota; however, they may also affect non-targeted bacteria (Hirano et al., 2021; Maier et al., 2021). Therefore, it is necessary to analyze the effects of certain molecules on individual bacteria. As our method makes it possible to culture a wide range of commensal intestinal bacteria under the same conditions, it may be useful for future research on agents that improve the intestinal microbiota.

## Data availability statement

The original contributions presented in the study are included in the article/**Supplementary Material**. Further inquiries can be directed to the corresponding author.

## Author contributions

Conceptualization, RH and SK; Data curation, RH; Methodology, RH, IN, RS, and SK; Investigation, RH, IN, RN, AB, and RS; Validation, AB; Resources, SK; Writing – Original Draft, RH and SK; Writing – Review and Editing, RH, and SK; Visualization, RH; Funding Acquisition, SK; Project Administration, SK; Supervision, SK. All authors contributed to the article and approved the submitted version.

## Footnotes

1
https://www.jcm.riken.jp/cgi-bin/jcm/jcm_grmd?GRMD=14
