# Supplementary figures and images for: Development of culture methods capable of culturing a wide range of predominant species of intestinal bacteria
Source: Front Cell Infect Microbiol. 2023 Jul 13;13:1056866. doi: 10.3389/fcimb.2023.1056866 (PMC10374021; doi:10.3389/fcimb.2023.1056866)

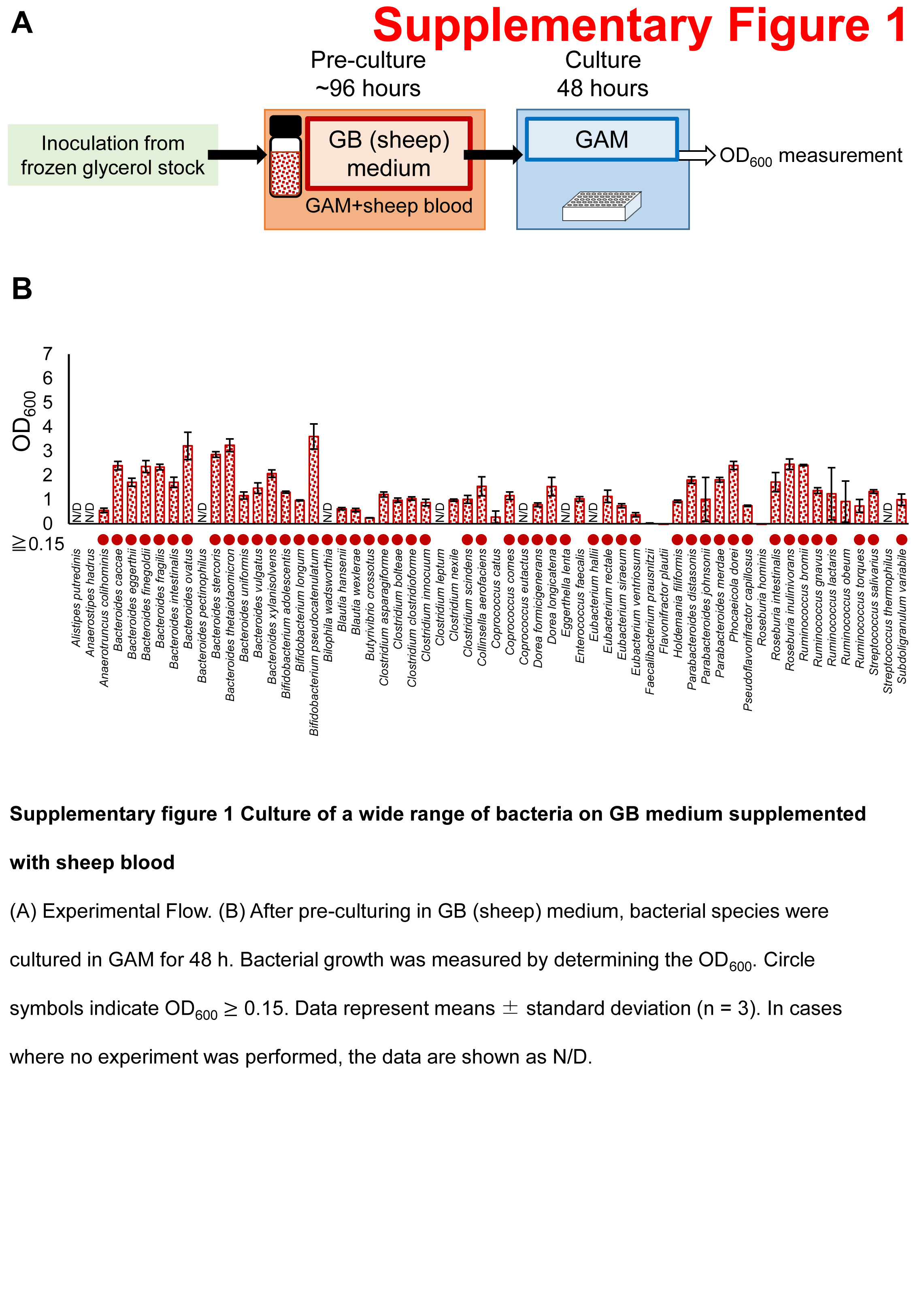

Supplement: Supplementary file 1 [file Image_1.jpeg]

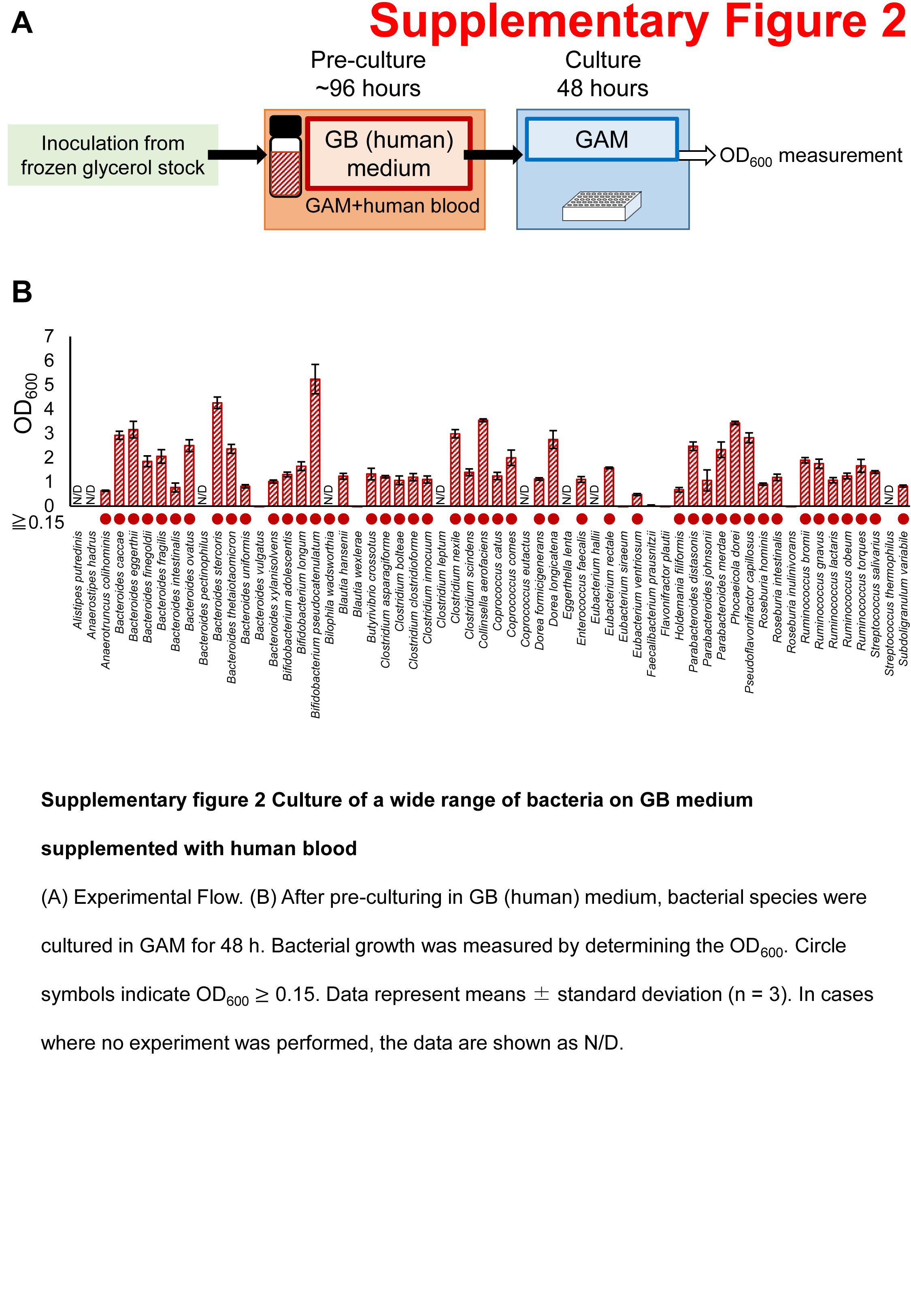

Supplement: Supplementary file 2 [file Image_2.jpeg]
